# Revisiting food insecurity gender disparity

**DOI:** 10.1371/journal.pone.0287593

**Published:** 2023-08-15

**Authors:** Andres Silva, Andres Astorga, Rodrigo Faundez, Karla Santos

**Affiliations:** 1 Escuela de Nutricion y Dietetica, Facultad de Ciencias para el Cuidado de la Salud, Universidad San Sebastian, Santiago, Chile; 2 Escuela de Economia, Facultad de Economia, Gobierno y Comunicaciones, Universidad Central de Chile, Santiago, Chile; University of Agriculture Faisalabad, PAKISTAN

## Abstract

Previous research has shown that woman-headed households, more than man-headed ones, experience food insecurity. The purpose of this article is to contribute on the determinants that are linked to this gender disparity. Using a nationally representative dataset from Chile, we found that food security household head gender disparity is associated with marital status (having or not a partner) and household composition (having children or seniors). In contrast, gender disparity is not strongly associated with household income and household head educational differences. In this way, we expect to bring evidence to inform new alternatives that help mitigate food security gender disparity.

## Introduction

Goal 2 of the Sustainable Development Goals is to eradicate hunger, achieve food security, improve nutrition and promote sustainable agriculture [1]. The term food security is currently defined by the Food and Agriculture Organization (FAO) as the physical, economic and social access, at all times, to sufficient, safe, and nutritious food to meet people’s dietary needs and food preferences for a healthy and active life [2]. Then, a household can experience food security, or a degree of lack of it, such as, mild, moderate or severe food insecurity.

Recently, the COVID-19 pandemic has increased the concern regarding food security, especially regarding economic and physical food access. In Latin America and the Caribbean, the COVID-19 pandemic has led to an increase of nine points of the population that experiences moderate or severe food insecurity [3]. The population that experiences moderate or severe food insecurity has increased from 207.0 to 267.2 million [3]. Therefore, the COVID-19 pandemic has enhanced the relevance of food security.

Food insecurity does not affect the population in the same way. Historically, the analysis on the determinants of food security has been dominated by household income, household head education and household composition. Previous research has shown that high-income households are less likely to experience food insecurity compared to low-income households [4]. It is expected that high-income households are less affected by economic fluctuations, food price changes or food shortage of any kind. In the same line, high-educated households are less likely to experience food insecurity compared to low-educated households [5, 6]. With respect to household composition, households with more children tend to experience more food insecurity than households without children [7, 8].

Previous evidence also shows that a larger proportion of woman-headed households experience food insecurity compared to man-headed households [8–10]. However, previous analyses have focused on food insecurity determinants. More recently, the COVID-19 pandemic has increased food insecurity disparities by gender [3]. In this sense, it is unclear whether the determinants that have been historically used to analyze food insecurity proportion are useful or not to disentangle food insecurity gender disparity.

The purpose of this article is to identify the determinants of the food insecurity gender disparity at the household level. Using the *Encuesta Nacional de Caracterización Socioeconómica de Hogares de Chile*, a nationally representative socioeconomic survey, we found that 16.2% of woman-headed households experience food insecurity, while 10.9% of man-headed households experience food insecurity, therefore; the food insecurity gender disparity is 5.3%. However, the gender disparity is not significant in one-member households. Rather than income or education differences, we found that marital status (having or not a partner) and household composition (having children or seniors) are associated with food insecurity disparity. Finally, our article suggests some new venues to consider in order to mitigate gender disparities in food security.

## Background

At the individual level, evidence shows that women are more likely to experience food insecurity than men [11, 12]. Using a worldwide data set and the Oaxaca-Blinder decomposition approach, Broussard found that women are 1.7–4.2 percent more likely to experience mild food insecurity and 2.0 percent more likely to experience severe food insecurity [11]. However, this study does not distinguish between one-member vs. multi-member households. In this way, we are not able to assess whether the gender disparity at the individual level can be associated to actual gender effect, or it is consequence of being a household member.

Additionally, women tend to make healthier food choices. Comparing data from college students from 23 countries, women are more likely than men to report avoiding high-fat foods, eating fruit and fiber and limiting salt consumption [13]. According to the authors, this trend is mainly explained by weight control rather than a stronger belief regarding healthy eating. In Canada, Colapinto *et al.*, found that women are more likely to eat fruit and vegetable more frequently than men [14]. Nevertheless, women, more than men, believe in the relevance of healthy eating. Also, the stronger preferences for healthy food choices in women may be driven by ethical concerns on food production, such as animal welfare [15]. According to Placzek, while men are influenced by taste, women that make healthy food choices are led by quality, price and family preferences [16]. Besides, women are more likely than men to consume the recommended amount of fruit and vegetable [14]. In a more general way, controlling for food insecurity, Graham *et al.,* found that men are less likely to report higher life satisfaction than women, which highlight gender perceptions differences [12].

At the household level, Quisumbing *et al.,* stated that women can contribute to food security through (i) home food procurement, (ii) improving economic access, additional household income, and (iii) time and cooking skills to prepare food [17]. Regarding home food procurement, such as gardening, fishing, foraging, backyard livestock and canning, Niles *et al.,* found a possible effect on food security, while they may be a relevant safety net for food insecure households [18]. However, only in food secure ones, food procurement activities are associated with higher fruit and vegetable intake.

The household income effect would also depend on the household composition that is discussed later in this article. Basically, additional household income would occur when the woman is not alone, with a partner, in charge of the household. Finally, cooking skills are associated with a wider variety of food consumption. It is expected that women, who have historically developed stronger cooking skills than men, would have better food security. Nunnery *et al.*, using a sample of low-income women, found that, through home availability of a variety of fresh fruit and vegetables, food security is associated to daily fruit and vegetable intake [19].

Despite the contributions that women make to household food security presented by Quisumbing *et al.*, previous research has shown that woman-headed households are more likely to experience food insecurity compared to man-headed households [9, 17, 20, 21]. Using the Oaxaca-Blinder decomposition approach, in a rural setting in Ethiopia, Gebre *et al.* found similar results using a different gender definition [10]. Comparing gender of the agricultural decision-making households instead of gender of the household head, Gebre *et al.* found that woman decision-making households had a larger probability of experiencing food insecurity [10]. Magaña-Lemus *et al.*, in Mexico, using national representative dataset, found that woman-headed households were 2.5% more likely to experience food insecurity [21].

Finally, social capital may also play a relevant role in food security. According to Willis, social capital is a representation of less tangible social resources that can link individuals with tangible resources [22]. In the case of food security, the least tangible resources would be social ties with friends, family or neighbors, as well as participation in organizations and churches. This link can be direct (giving away or sharing food) or indirect (through money to buy food or make trips to the supermarket).

Social capital involves more than one dimension: Lee *et al.*, found that a household with neighbors to rely on is less likely to experience food insecurity [23]. Using focus group analysis with rural older adults, Valliant *et al.*, and also Sharpe *et al.*, found that transportation (providing a car to go shopping and attend care centers) is a relevant dimension of social capital on food security [24, 25].

As a result, previous evidence shows that social capital contributes to improving household food security [26, 27], in contrast, the lack of social capital is associated to a higher probability of lack of food [28]. In concrete, de Sousa *et al.* found that low-social-capital households were twice more likely to experience food insecurity [29]. Regarding the determinants of social capital, according to Perkins *et al.*, the household head gender plays a role in the social capital, and then, in food security [30].

Therefore, at the individual level, women tend to choose a healthier food basket, and contribute to their households with procurement, cooking skills and time. Broussard found that, at the individual level, household income, education and social network are relevant determinants [11]. Nevertheless, woman-headed households are more likely to experience food insecurity [9, 20, 21]. In this context, our research analyzes determinants that can help to decompose the food insecurity gender disparity.

## Materials and methods

### Methodology

Taking into account that the purpose of this article is to identify the determinants of the food insecurity gender disparity at the household level, we analyzed the effect of women in three ways: (i) comparing graphically, and using descriptive statistics, the proportion of women (number of women divided by household size) per household head gender, (ii) comparing graphically how household food insecurity changes with an additional adult woman, and (iii) using household head gender as explanatory variable in probit and mediation models.

### Data set

The *Encuesta Nacional de Caracterización Socioeconómica de Hogares de Chile*, known as CASEN Survey (National Socio Economic Household Characterization Survey) has been carried out since 1987 with a biannual or triannual periodicity by the Government of Chile through the Ministry of Social Development and Family. The CASEN Survey, collected in-person, is representative at rural/urban, regional and national levels. In 2017, the survey considered data from 70,677 households, equivalent to 216,439 people. After data weighting, this corresponds to 5.8 million households [31].

The objective of this survey is to have information that allows to periodically know the situation of the households and the population in relation to demographic aspects, education, housing, composition of the household, health, work and income. The CASEN Survey is used for estimating the magnitude of poverty and income distribution; identify the deficiencies and demands of the population in the indicated areas; and evaluate the different disparities that separate the different social segments and territorial areas. It also evaluates the impact of the social policy estimating its coverage, the targeting and the distribution of the fiscal expenditure of the main social programs of national scope among households and according to their income level. The anonymized data set, and support documentation are publicly available at http://observatorio.ministeriodesarrollosocial.gob.cl/encuesta-casen-2017.

The indicator 2.1.2 of the Sustainable Development Goals defines the Food Insecurity Experience Scale (FIES) and captures the dimension related to food and the difficulty of accessing it. Using a set of eight questions that are sorted in terms of food insecurity severity, the FIES measures the severity of food insecurity at the household or individual level. The details of the FIES can be found at Ballard *et al.* [32]. The FIES questionnaire has been internationally validated and implemented in several countries around the world.

By containing items from a survey, for the FIES, the Rasch model allows examining the psychometric properties of the tool, applying the principles of the Item Response Theory [33]. The Rasch model, which is used for binary instruments, defines the probability of an affirmative response (“yes”, for the FIES) in each of the eight items, based on the positional differences between the individual’s response and the location of the item [34].

Since the wave 2017, the CASEN includes the FIES questionnaire, which allowed us to combine socio-demographic and food insecurity data. We used the data set to estimate the proportion of the population that experiences moderate or severe food insecurity. Following the procedure presented by Viviani, we converted the FIES questionnaire answers into parameters associated to the probability of experiencing moderate or severe food insecurity [35]. The procedure can be summarized in these steps:

- We coded the answers in a binary way (yes—no) and excluded from the analysis the answers “does not know” and “does not answer”. The raw score corresponds to the sum of affirmative responses (yes) to each of the eight questions. Therefore, the higher the score, the more likely that the respondent experiences food insecurity.
- We calculated the parameters of the items to express food insecurity and thus assigned the position of the items according to the parameters, based on the general pattern of responses, in a relative scale of severity. Thus, an item that represents a less severe experience of food insecurity will have a lower parameter value, and vice versa.
- We compared, using the equating function, the CASEN item parameters with the Standard Global vector, to later generate the probability of experiencing food insecurity per household. To ensure the quality of the data, we performed a statistical evaluation that allowed us to determine how well the FIES works in the population.

Following Endersan, we conducted a statistical validation using: (1) Infit, which contains values between 0.7 and 1.3, which means that there is an adequate fit in each item, that is, they worked well in the population; (2) Reliability, gives a value of 0.76, so it is considered an acceptable reliability, taking into account the number of items; and (3) Correlation matrix, with item values less than 0.4. Finally, we converted the food insecure variable as a binary variable [36]. We classified as food insecure those households with a probability of 0.5 or more, and the remaining as food secure. In this way, we obtained the food insecurity status as an additional variable in the CASEN data set.

### Model

Mediation analysis aims to quantify the effect of an independent variable on a dependent variable through a third variable or mediating variable [37]. A mediating variable is one that is inserted in a causal sequence between two variables [38]. Therefore, mediation analysis helps to empirically test a theoretical explanation [39]. We conducted mediation analysis using the user-written command developed by [40].

In our case, firstly, following previous research, we used a probit model to link households’ characteristics with food insecurity. Then, we used meditation analysis to disentangle the relationship between household head gender and food insecurity in a direct path and an indirect path. The direct path is the straight forward relationship between household head gender and food insecurity. The indirect path is the relationship between household head gender and food insecurity that goes through another variable, which is called mediator. In our study, in alternative specifications, we used the following variables as mediators: (i) household income, (ii) household head education and (iii) marital status.

Household income has been historically pointed out as a food insecurity determinant [4]. Also, household head education has been indicated as a food insecurity determinant [5, 6]. Since these two determinants can be also associated to gender disparities, we used them as potential mediators. Finally, we used marital status as a way to analyze how internal relationships at the household can be associated to food insecurity. As control variables, we included household composition, zone (urban/rural) and household-head age. Then, we repeated the analyses using different sub-samples of social capital variables (car and money availability for an emergency). In this way, we were able to show whether household-head gender was associated to a food insecurity gender disparity.

### Descriptive statistics

Tables 1 and 2 compare descriptive statistics by household head gender. Each average is compared using a linear regression. Using representative weights, the dependent variable is the variable in the first column, and the independent variable is the household head gender indicator. The t-value and p-value of the estimated coefficients are presented in the last two columns.

**Table 1:** Overall descriptive statistics by household head gender.

	Man Headed Household		Woman Headed Household		Difference	
	Mean	SE	Mean	SE	t-value	p-value
age household head, years	52.716	(15.946)	53.901	(17.077)	9.50	0.000
marital status household head, couple	0.786	(0.410)	0.265	(0.441)	-162.06	0.000
**Education Household Head**						
elementary and mid school	0.287	(0.452)	0.321	(0.467)	9.91	0.000
high school	0.427	(0.495)	0.415	(0.493)	-3.19	0.001
college	0.259	(0.438)	0.241	(0.428)	-5.49	0.000
graduate school	0.026	(0.160)	0.022	(0.146)	-3.81	0.000
**Income Category**						
very low income	0.057	(0.232)	0.100	(0.300)	21.40	0.000
low income	0.241	(0.428)	0.323	(0.468)	24.32	0.000
low-medium income	0.226	(0.418)	0.236	(0.425)	3.34	0.001
medium income	0.149	(0.356)	0.128	(0.334)	-7.91	0.000
medium-high income	0.158	(0.365)	0.113	(0.316)	-17.32	0.000
high income	0.072	(0.259)	0.050	(0.218)	-12.07	0.000
very high income	0.097	(0.296)	0.050	(0.217)	-23.49	0.000
**Household Composition**						
number of men at the household	1.748	(0.927)	1.072	(1.042)	-91.15	0.000
number of women at the household	1.486	(1.084)	1.782	(0.980)	37.47	0.000
number of children at the household	0.721	(0.988)	0.709	(0.975)	-1.66	0.096
number of adult men at the household	1.135	(0.759)	0.641	(0.772)	-85.05	0.000
number of adult women at the household	0.976	(0.776)	1.128	(0.778)	25.80	0.000
number of seniors at the household	0.402	(0.706)	0.376	(0.586)	-5.25	0.000
**Type of Household**						
urban	0.859	(0.348)	0.913	(0.282)	22.28	0.000
rural	0.141	(0.348)	0.087	(0.282)	-22.28	0.000
**Category Regions**						
metropolitan	0.398	(0.490)	0.399	(0.490)	0.09	0.932
north	0.117	(0.322)	0.124	(0.329)	2.51	0.012
center	0.340	(0.474)	0.338	(0.473)	-0.71	0.479
south	0.144	(0.351)	0.140	(0.347)	-1.49	0.135
**FIES Questionnaire Results**						
prob. moderate or severe food insecurity	0.109	(0.311)	0.162	(0.368)	20.77	0.000
Observations	41,450		29,498			

Note: The differences are computed using a linear regression of the variable (as dependent variable) and a dummy to indicate household head gender. An adult corresponds to someone between 18 and 65 years old. A person older than 65 years old is a senior and a person younger than 18 years old is a child. There are 705 missing values out of 70,948 observations in the FIES responses, which represents 1.0% of the data set.

**Table 2:** Social capital and job descriptive statistics by household head gender.

	Man Headed Household		Woman Headed Household		Difference	
	Mean	SE	Mean	SE	t-value	p-value
household size	3.234	(1.577)	2.854	(1.539)	-32.08	0.000
working hours/week	45.607	(13.632)	39.850	(15.824)	-41.03	0.000
**Type of Job, formality**						
formal job, percent	0.489	(0.500)	0.329	(0.470)	-43.37	0.000
number of women with formal job	0.310	(0.526)	0.460	(0.602)	35.41	0.000
number of men with formal job	0.639	(0.660)	0.295	(0.542)	-73.85	0.000
number of women with informal job	0.063	(0.253)	0.110	(0.333)	21.56	0.000
number of men with informal job	0.099	(0.326)	0.059	(0.257)	-17.59	0.000
**Length of Service, current job**						
less than 2 years	0.255	(0.436)	0.294	(0.456)	9.06	0.000
3 to 5 years	0.176	(0.381)	0.236	(0.425)	15.70	0.000
6 to 10 years	0.173	(0.378)	0.181	(0.385)	2.11	0.035
more than 11 years	0.397	(0.489)	0.289	(0.453)	-23.13	0.000
**Institutional Networks**						
number institutions (any member)	0.798	(1.120)	0.617	(0.900)	-23.05	0.000
**Social Ties**						
help in case of sickness	0.893	(0.309)	0.887	(0.317)	-2.80	0.005
help to take care handicap or children	0.835	(0.371)	0.821	(0.383)	-4.79	0.000
have someone who can lend a car	0.858	(0.349)	0.807	(0.395)	-18.13	0.000
have someone who can lend money for an emergency	0.794	(0.404)	0.764	(0.425)	-9.65	0.000
legal advice/help	0.746	(0.435)	0.723	(0.448)	-7.06	0.000
tech advice/help	0.845	(0.362)	0.819	(0.385)	-9.16	0.000
home repair help	0.810	(0.392)	0.734	(0.442)	-24.10	0.000
help to find a job	0.626	(0.484)	0.576	(0.494)	-13.46	0.000
help advice	0.831	(0.375)	0.810	(0.392)	-7.09	0.000
help with another language	0.510	(0.500)	0.473	(0.499)	-9.65	0.000
access to someone with a college degree	0.730	(0.444)	0.711	(0.453)	-5.61	0.000
Observations	41,450		29,498			

Note: The differences are computed using a linear regression of the variable (as dependent variable) and a dummy to indicate household head gender. An adult corresponds to someone between 18 and 65 years old. A person older than 65 years old is a senior and a person younger than 18 years old is a child.

In Table 1, in terms of education level, man-headed households are slightly more educated than woman-headed households. However, we do not have information related to the type of college degree. The gender disparity is stronger in terms of household income. For instance, 32.7% of man-headed households and 21.3% of woman-headed households have medium-high income or more. In terms of household composition, man-headed households, compared to woman-headed households, have 0.3 more adults. Considering that 78.6% of the man-headed households live as a couple, instead of 26.5% of woman-headed households. As a result, a couple in charge of the household, instead of a single person, would represent a relevant difference in terms of time allocation. Finally, in most of cases, region does not show significant differences.

The proportion of households experiencing moderate or severe food insecurity is presented at the bottom of Table 1. The proportion of the population that experiences moderate or severe food insecurity is relevant and significantly larger in woman-headed households compared to man-headed households. Using statistical survey weights, 16.2% of woman-headed households and 10.9% of man-headed households experience food insecurity, which translates into a gender disparity of 5.3%. The latter value is the reference point for the results of the probit and mediation analysis.

S1 Fig, in the Supplemental Information Section, shows how food insecurity changes with an additional adult (woman on top figure, man at the bottom figure) by household head gender. We define an adult as a person between 18 and 65 years old. In a household with no adult woman, man-headed and woman-headed experience similar levels of food insecurity (close to 11%). Then, after adding subsequently adult women, food insecurity in man-headed households are stable around 11%, while woman-headed households are stable around 18%. After the first adult woman, adding adult women does not seem to be related to food insecurity levels. In a household with no adult man, 17.0% of woman-headed experience food insecurity and 8.1% man-headed ones. Then, after adding subsequently adult men, food insecurity in man-headed households are relatively stable around 11–13%, while woman-headed households are relatively stable around 15%. After the first adult man, adding adult men does not seem to be related to food insecurity levels. Therefore, per gender, after the first one, an additional adult does not seem to be related with changes in the proportion of households that experience food security.

Following the same logic, S2 Fig shows how food insecurity changes after adding children subsequently. In contrast with figures for adults, especially for woman-headed households, with one child, having an additional child increases relevantly the proportion of households that experience food insecurity. Specifically, the 12.8% of woman-headed households without children experience food insecurity, 18.2% of woman-headed households with a child, 21.3% of woman-headed households with two children, and 28.5% of woman-headed households with three children. For man-headed households, there is a softer and later increase, from 9.9% to 15.8%, in the proportion of households that experience food insecurity.

CASEN (2017) also has variables that are uncommon on food security data sets. Table 2 shows the descriptive statistics of working hours of the household head, degree of job formality (having a job contract), how long the household head has worked in the current job, and social capital: institutional network and social ties. Institutional network corresponds to the participation of a household member in a church, neighbor association or another thematic social organization. Finally, having social ties means that the households have someone that can help in case that is needed, for instance, to take care of children, provide legal support or help to find a job.

According to Table 2, 48.9% of man-headed households have one formal job, while 32.9% of woman-headed households have one. In the same way, man-headed households have more formal workers, workers with a job contract, compared to woman-headed households. Both types of households have a similar number of informal workers. These differences in terms of job contract can be associated to household size. The fact that man-headed households have a larger household size can be linked to having more adults and more men at the household. These results are also aligned to Table 1 that shows that man-headed households have higher income.

Also linked to job formality, 57.0% of man-headed households, compared to 47.0% of woman-headed ones, have six or more years at the current job. We cannot identify a causal path, however; it is clear that man-headed households have more formal jobs, work more hours per week, stay on that job for a longer period of time and have a higher income. These job-related determinants may also explain, at least in some extent, the food security gender disparity.

Finally, the CASEN data set includes questionnaires on social capital: institutional network and eleven types of social ties. According to Broussard, individuals with social capital are less likely to experience food insecurity [11]. Man-headed households, on average, participate in 0.8 institutions, while woman-headed households participate in 0.6 institutions. Comparing the eleven types of available help, social ties, a larger proportion of man-headed households have social ties compared to woman-headed households. This may be explained by the larger proportion of man-headed households who live with a partner, in other words, two adults in charge of the household. It is expected that a household that is active has social ties that provide support to deal with food insecurity, compared to an isolated household. In the best of our knowledge, few articles take into account social capital determinants. These social ties related determinants may also explain, at least in some extent, the food security gender disparity.

## Results and discussion

### Mean comparison

Table 3 compares mutually exclusive groups of man-headed and woman-headed households, based on income (low and high), composition (with or without children, and one member or multiple-member, with or without seniors). We found that one-member households and high-income households without children do not have a significant food insecurity gender disparity. In contrast, having children, having seniors and low-income are household characteristics that increase food insecurity gender disparity. As reference, 13.1% of the population experiences moderate or severe food insecurity. As presented in Table 1, 10.9% of man-headed households and 16.2% of woman-headed households experience moderate or severe food insecurity, which represents a food security gender disparity of 5.3%.

**Table 3:** Unconditional mean comparison moderate and severe food insecurity.

	Man Headed Household		Woman Headed Household		Difference	
	Observations	Mean	Observations	Mean	t-value	p-value
low-income households without children	17,268	0.127	13,715	0.151	-7.250	0.000
high-income households without children	6,053	0.033	3,006	0.041	-2.338	0.019
low-income households with children	11,970	0.161	9,980	0.244	-15.394	0.000
high-income households with children	5,749	0.046	2,502	0.078	-5.556	0.000
one-member households	5,303	0.132	5,818	0.128	0.634	0.526
multiple-member households	35,737	0.098	23,385	0.156	-23.783	0.000
households with seniors	29,112	0.110	19,143	0.175	-22.860	0.000
households without seniors	11,928	0.085	10,060	0.104	-5.493	0.000

Note: Data presented in the table without statistical survey weights. As reference, the gender disparity is 5.3% (= 16.2%—10.9%) using statistical survey weights and 4.6 (=15.0%—10.3%) without statistical survey weights. One-member households do not have children.

Table 4 shows the percentage of population that experiences moderate or severe food insecurity by different groups based on income and household composition. One-member households are 15.4% and multi-member are 84.6% of the full sample (considering statistical weights). The result of the full-sample is weighted average of the results presented for each sub-sample. The first row shows the marginal effect without control variables, while the second row shows the marginal effect controlling for household size. Household composition, as a control variable, is included as a set of dummy variables on number of children, adults and seniors at the household. Finally, the third row shows the marginal effect controlling for household composition, zone (urban vs. rural) and household head age. After a probit model, it shows that the household head gender marginal effect is 5.3%. This household head gender disparity drops 0.8 percent point after controlling for household composition at the household.

**Table 4:** Household gender probit marginal effects.

Control Variables	Sub-samples				Full Sample	
	One-member		Multi-members			
none	-0.018	(0.001)	0.065	(0.000)	0.053	(0.000)
household composition	-0.006	(0.001)	0.055	(0.000)	0.045	(0.000)
household composition, zone, hh age	-0.006	(0.001)	0.055	(0.000)	0.045	(0.000)
Observations	11,121		59,122		70,243	

Note: Marginal effect after alternative probit specifications and sub-samples using statistical survey weights. Standard errors in parentheses. Household composition is included as a set of dummy variables on number of children, adults and seniors at the household. One-member households are 15.4% and 84.6% are multi-member ones.

Controlling for household composition does not capture the difference between one-member and multiple-member households. In summary, 4.5% out of 5.3% of food insecurity gender disparity is not associated to household composition, zone, or household head age. Now, the question would be whether the food insecurity gender disparity is mediated by household income, household head education, and marital status. In the coming section, using mediation analysis, we will decompose the association of household head gender and food insecurity in direct and indirect paths, through a set of alternative mediators.

### Mediation results

Table 5 shows the mediation probit results using alternative mediators and keeping household gender as an explanatory variable and food insecurity as an outcome variable. Since the mediators and the outcome are binary variables, we need to use probit to model the data. The complete probit output is available by request. Columns 2 and 3 show the effect using education as mediator, columns 4 and 5 show the results using household income as mediator, and finally, columns 6 and 7 show the result using marital status as mediator.

**Table 5:** Mediation analysis results using probit.

	Education		Income		Marital Status	
Effect	Mean	Conf Interval	Mean	Conf Interval	Mean	Conf Interval
Average Mediation	0.002	[0.002 0.003]	0.005	[0.004 0.005]	0.017	[0.014 0.020]
Average Direct Effect	0.040	[0.035 0.045]	0.037	[0.032 0.042]	0.020	[0.015 0.026]
Total Effect	0.042	[0.037 0.047]	0.042	[0.037 0.047]	0.037	[0.032 0.042]
Total Effect Mediated (%)	0.050	[0.045 0.057]	0.116	[0.104 0.132]	0.458	[0.403 0.529]

Note: Bootstrapping results, after 1,000 replications, in parentheses. Columns 2 and 3 show the mediation probit results using household head education as a mediator. Columns 4 and 5 show the mediation probit results using household income as a mediator, and similarly, columns 6 and 7 show the results using marital status as a mediator.

The purpose of the mediation model is to test whether the household head gender, indirectly through the mediation path or directly, is associated to changes in food insecurity. The mediation path is decomposed into two portions and each one is represented by a probit equation. The first mediation equation tests whether household head gender is linked with changes on the mediation (income, education or marital status). The second mediation equation tests whether the mediator, one at the time, is linked with changes in food insecurity.

Then, we conducted a bootstrapping to estimate confidence intervals. Table 5 shows that the total effect is between 3.7% and 4.2%, which adds the direct and indirect/mediated paths. Then, 5.0% of the total effect is mediated by household head education, 11.6% is mediated by household income and 45.8% is mediated by marital status. In amount, the mediation associated to marital status is, at least, three times larger than the one mediated by household income or household head education.

We analyzed how social ties would change the marital status mediated share. Table 6 shows the results using two sets of mutually exclusive household groups using social ties criteria: having someone to borrow a car from and having someone to borrow money from. As presented in columns 2 to 5, with respect to having someone to borrow a car from in case of an emergency, in average, marital status mediates 66.2% for households that have someone, while the percentage drops to 40.2% for households that do not have someone. As presented in columns 6 to 9, a similar trend happens with respect to having someone to borrow money from, the mediated share is larger in case of having someone compared to those that do not have someone to borrow money from.

**Table 6:** Mediation analysis results using marital status as mediation by social ties.

	No Car		Car		No Money		Money	
Effect	Mean	Conf Interval	Mean	Conf Interval	Mean	Conf Interval	Mean	Conf Interval
Average Mediation	0.023	[0.016 0.031]	0.014	[0.011 0.018]	0.034	[0.028 0.041]	0.014	[0.011 0.018]
Average Direct Effect	0.012	[-0.006 0.029]	0.021	[0.015 0.028]	0.028	[0.013 0.042]	0.020	[0.013 0.027]
Total Effect	0.035	[0.019 0.051]	0.035	[0.030 0.041]	0.061	[0.048 0.075]	0.035	[0.029 0.041]
Total Effect Mediated (%)	0.662	[0.455 1.210]	0.402	[0.344 0.482]	0.554	[0.458 0.711]	0.417	[0.354 0.503]

Note: Bootstrapping results, after 1,000 replications, in parentheses. The models use marital status as mediator to create two mutually exclusive sub-samples. Columns 2 to 5 show the mediation probit results using the variable “having someone to borrow a car from” in case of an emergency to create sub-samples. Columns 6 to 9 show the mediation results using “having someone to borrow money from” in case of an emergency to create sub-samples.

In summary, in one-member households without control variables, Table 4 shows that woman-headed households experience less food insecurity than man-headed ones, while the opposite happens for multi-member households. According to Table 4, food insecurity gender disparity moves from 5.3% to 4.5% after controlling for household composition, zone (urban/rural) and household head age. Then, in Table 5, the mediation analysis shows that household head gender is associated to a total effect of 3.7%, from which 1.7 percent point is mediated by marital status (indirect effect) and the remaining 2.0 percent points are classified as direct effect.

### Discussion

In Chile, as presented by [9], among households with children, we found that a larger proportion of woman-headed households experience food insecurity. As presented in Table 3, having children and low-income, having a senior and multiple-member households are determinants that increase the food insecurity gender disparity. According to [6], in the same line that Figure in the Appendix, the number of children at the households has a larger effect on woman-headed households than on man-headed households.

In one-member households, we found that being a woman is associated with 1.8% less likelihood of experiencing food insecurity, and 0.6% are less likely after controlling for household composition, zone and household-head age. Giacoman *et al.* found that one-member households of older women experience significantly less food insecurity compared to households with older men alone [9].

However, the household head gender marginal effect changes in a relevant way in one-member households compared to multiple-member households. Nord found that one-member households have a larger proportion of food insecurity [41]. As presented in Table 3, in the case of man-headed households, we found that a larger proportion of one-member households experience food insecurity, while the opposite happens on woman-headed households. Therefore, in multiple-member households, a larger proportion of woman-headed households experience food insecurity.

Using individual-data from Malawi, Brunelli *et al.* found that gender effect becomes non-significant after controlling for education and income, which suggests that food insecurity disparities are associated to educational and income differences rather than unobserved determinants [42]. However, food security determinants may vary considering individuals vs. household levels [11]. A food secure household does not imply that each member is food secure. Due to time use and gender privileges, Hadley *et al.* found that girls are more likely than boys to experience food insecurity in a food insecure household [43]. We found that a food insecurity gender disparity is relevant at the household level, especially, in woman-headed households with children.

Household-head education and household income have been pointed out as key food security determinants. However, we found that they are not necessarily relevant to analyze food insecurity gender disparity. Moreover, household income can be studied beyond its level. A formal employment would offer more income stability to a household, which would also contribute to food security. As presented by Gundersen, employment and income are not likely to be exogenous determinants to explain food security status [44]. In Table 2, we briefly discuss some aspects regarding job formality. However, this discussion can be taken further in future research.

Another possible determinant to explain gender difference is time allocation. Table 2 shows that, on average, man-headed households work 5.8 hours per week more than woman-headed households. This different time allocation may be led by a large proportion of women without a partner. In other words, women need to divide their time into household duties and job duties since they do not have a partner to rely on. According to the ENUT (*Encuesta Nacional del Uso del Tiempo*, which translates to National Survey of Time Use), conducted by INE (*Instituto Nacional de Estadísticas*, which translates to National Statistics Institute), men spend 5.7 hours/day in paid-work and 2.6 hours/day in non-paid work, while women spend 3.6 and 5.8 hours respectively. The time-allocation differences can also be the result of marital status and the number of children, the latter two have pointed out a relevant determinant to explain food security [6].

Also, linked to the use of time is job characteristics in terms of formality (with o without contract) and job length, and then, to salary [45]. A formal job is a job with a legal contract. Formal jobs tend to be better paid than informal jobs [46]. Some of these job characteristics can be linked with education attainment. Previous research has shown that being employed and earning a higher income led to lower chances of experiencing food insecurity [44]. Being in the workforce does not necessarily cause food security. According to Nord, 61% of households with very low food security among children, have one or more members employed full-time [41]. In this sense, job formality, rather than only being part of the workforce, may be playing a relevant role on food security [47].

Joshi *et al.*, in India, found that informal work, as well as lack of basic health and hygiene facilities, are food insecurity predictors [48]. A legal job contract imposes duties to the employee and employer relationship. In Chile, a legal job contract includes an unemployment insurance, retirement contribution and health coverage that provide, in some extent, more welfare to households compared to informal workers. Social protection services offer households protection against food insecurity, having an effect on human capital and improving access and stability to safe and nutritious food [49].

Finally, food insecurity gender disparity would be larger in woman-headed households without a partner and weak social ties. Consistent with Broussard, we also found that household heads with a partner are less likely to experience food insecurity at the household [11]. We found such effect only in low-income households with children, multiple-member households and households with seniors.

## Conclusions

Considering the descriptive statistics of Table 1, man-headed households are more likely to experience food security and have a relevant and significant larger proportion of household heads living with a partner at the household. Therefore, our results suggest that having a partner, rather than household income or household head education, plays a relevant role for multi-member households. Finally, Table 6 shows that marital status, rather than household head education and household income, mediate food insecurity head gender disparity. In this context, this mediation is stronger for household with weak social ties (in terms of having someone to borrow money or a car from). In other words, in isolated multi-member households, it is particularly relevant that the household head has a partner to mitigate food insecurity gender disparity.

We found that, food insecurity gender disparity, as a proportion of the population that experiences moderate or severe food insecurity, is associated to household composition, rather than due to an individual characteristic. Marital status plays a more relevant role in the food insecurity head gender disparity in the case of weak social ties. Our results suggest that woman-headed households with weak or no social ties need to rely more on having a partner to avoid a larger food insecurity head gender disparity.

The food security gender disparity is a global phenomenon. Household composition needs to be taken into account to tackle the gender disparity of food insecurity. Food policy to mitigate food insecurity disparity would need to be mainly targeting woman-headed low-income households with children. For instance, Nunnery showed that the use of canned and frozen fruit and vegetable may improve food environment and dietary habits among low-income pregnant women [19]. Finally, food procurement is not enough to reduce food insecurity. Education [6] and social capital [19] need to be part of an effective food security strategy. Social capital variables can also be further analyzed. We expect that our research has helped to show the relevance of considering social capital, household composition (one member vs. multiple members) and marital status as part of the food policy debate.

## Supporting information

S1 Fig Changes in food insecurity percentage with additional adults. The data presented in the figures use statistical survey weights. Food insecurity column corresponds to the percentage of the population that experiences moderate or severe food insecurity. Woman share column corresponds to the percentage of women (adding female children, female adults and female seniors) divided by household size (sum of men and women) at the household.
(PDF)

S2 Fig Changes in food insecurity percentage with additional children. The data presented in the figures use statistical survey weights. Food insecurity column corresponds to the percentage of the population that experiences moderate or severe food insecurity. Woman share column corresponds to the percentage of women (adding female children, female adults and female seniors) divided by household size (sum of men and women) at the household.
(PDF)

S1 Dataset https://observatorio.ministeriodesarrollosocial.gob.cl/encuesta-casen-2017. (TXT)
