# Supplementary material for: Revisiting food insecurity gender disparity
Source: PLoS One. 2023 Aug 15;18(8):e0287593. doi: 10.1371/journal.pone.0287593 (PMC10426994; doi:10.1371/journal.pone.0287593)
Supplement: S1 Fig — The data presented in the figures use statistical survey weights. Food insecurity column corresponds to the percentage of the population that experiences moderate or severe food insecurity. Woman share column corresponds to the percentage of women (adding female children, female adults and female seniors) divided by household size (sum of men and women) at the household. (PDF) [file pone.0287593.s001.pdf]

Supporting information

S1 Fig 1. Changes in Food Insecurity Percentage with Additional Adults

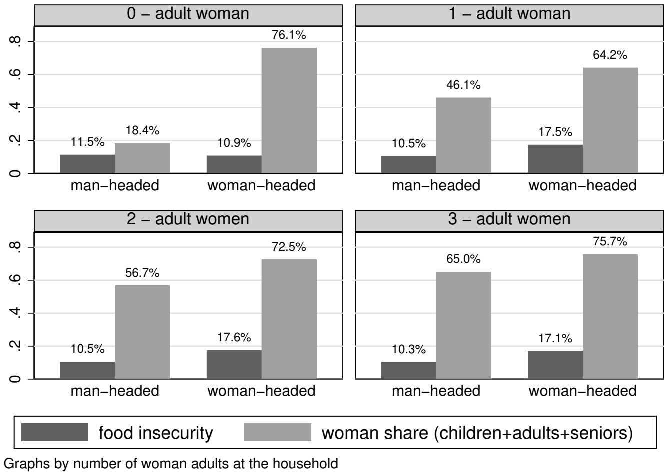

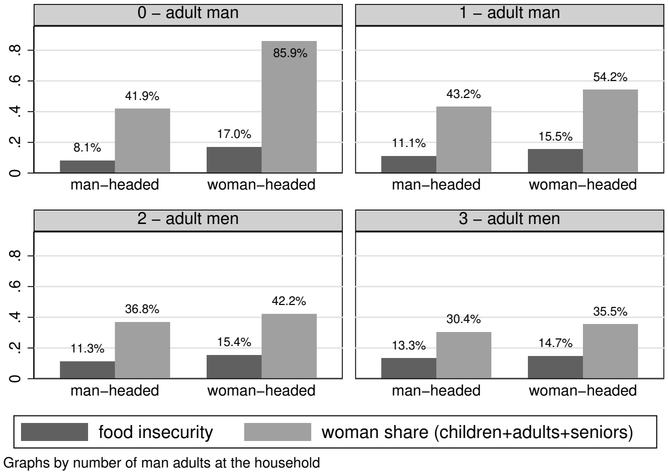

Note: The data presented in the figures use statistical survey weights. Food insecurity column corresponds to the percentage of the population that experiences moderate or severe food insecurity. Woman share column corresponds to the percentage of women (adding female children, female adults and female seniors) divided by household size (sum of men and women) at the household.
